# Queer Definitions in Hospital Diet

**Published:** 1911-09-23

**Authors:** E. W. Morris

**Affiliations:** Secretary. London Hospital, Whitechapel, E.


*To the Editor of* The Hospital.

### "QUEER DEFINITIONS IN HOSPITAL DIET."

*To the Editor of* The Hospital.

Sir,—I have read with very great interest your account of Mr. Conrad Thies' interview with the *Sunday School Chronicle* [The Hospital, September 9, page 602].

I should not trouble you with a letter, except for the fact that at the end of your quotation you refer to the practice of the London Hospital of asking its patients to provide their own tea, sugar, and butter. As Mr. Sydney Holland is at present in the north of Scotland, and probably has not seen your reference to him, I am replying on his behalf.

I think my friend Mr. Thies has been indulging in the old practice of putting up straw men in order that we may have the pleasure of seeing him knock them down. He points out that in certain hospitals medical officers may order champagne and other luxuries, but may not order tea, sugar, and butter. Of course if the rule as to tea, sugar, and butter had been adopted because we considered them a luxury, and yet allowed other luxuries to be ordered, our position would be "indefensible." Unfortunately for Mr. Thies' rhetoric, that is not our reason, and so far as I have been able to discover in tracing back the minutes, it never was the reason.

I think we all agree that the patients who are now treated in the hospitals are not the same in social position as they were at the time when most of our hospitals were founded. They are of a higher social position. A great many of our patients are those who can afford an ordinary and simple illness, but cannot afford long expensive illnesses, especially those involving a surgical operation. I think the hospitals are doing perfectly right in acknowledging that the social position of their patients has changed, and in agreeing to admit patients for treatment who would not have been admitted in the days when medical and surgical treatment was less expensive.

But because a hospital offers to give a poor person treatment which that person cannot afford to buy—skilled nursing, expensive dressings, expensive medicine, expensive treatment—is it also the duty of the hospital to become an hotel as well as a hospital, and provide all food free? Opinions may differ in this matter. Some committees may say "We prefer to give everything," others say, "We will give in treatment what the patient cannot afford to pay for, but we do expect the patient to contribute towards articles of daily consumption." Whatever view the hospital committees may take, they each have a good deal to say on their side, and neither, I am convinced, would agree that their position was, as Mr. Thies said, "absolutely indefensible." Yours very truly,

London Hospital, E. W. Morris, Secretary.

Whitechapel, E., September 19.

